# Effects of direct oral anticoagulants vs. vitamin K antagonists on acute kidney injury in patients with atrial fibrillation: A systematic review

**DOI:** 10.3389/fcvm.2023.1068269

**Published:** 2023-01-26

**Authors:** Chengfa Ren, Yudan Zhao, Dehui Liu

**Affiliations:** ^1^Department of Nephrology, Ganzhou People's Hospital Affiliated to Nanchang University, Ganzhou, Jiangxi, China; ^2^Medical Department, Queen Mary School, Nanchang University, Nanchang, China

**Keywords:** direct oral anticoagulants, vitamin K antagonists, acute kidney injury, atrial fibrillation, systematic review

## Abstract

**Background:**

Patients with atrial fibrillation (AF) are routinely prescribed oral anticoagulants to prevent thromboembolism. Concerns regarding the efficacy and safety of oral anticoagulants, such as vitamin K antagonists (VKA) and direct oral anticoagulants (DOACs), arise for patients with non-valvular atrial fibrillation (NVAF) because of their widespread use in clinical practice. Even though there have been an abundance of studies on this topic, it is still not clear if DOAC users with NVAF have a lower risk of acute kidney injury (AKI) than warfarin users.

**Methods and results:**

We conducted electronic searches in PubMed, Embase, and the Cochrane Library to identify relevant studies for this systematic review. We included randomized clinical trials and observational studies that reported on the incidence rate, hazard ratio (HR), and 95% confidence interval (95% CI) of AKI in patients using oral anticoagulants. This systemic review included six observational studies and four randomized clinical trials (RCT). The overall results showed that DOACs were associated with a lower AKI risk than warfarin. However, for NVAF patients with severe renal dysfunction, DOACs may not have a reduced risk of AKI compared to warfarin.

**Conclusion:**

The overall results suggest that, except for edoxaban, patients using DOACs may experience a reduced risk of AKI. However, it is uncertain whether this is also the case for patients with severe renal dysfunction. Further research is needed to confirm the effect of DOACs on this population.

## Introduction

Atrial fibrillation (AF) is a common type of arrhythmia that affects many adults. It occurs when abnormal electrical signals in the heart, produced by an ectopic focus1 rather than the heart's normal pacemaker (called the sinus node), cause the heart to beat irregularly and too fast. The incidence and prevalence of AF have been on the rise due to an aging population and the ability to more accurately diagnose the condition (1). Therefore, there has been a greater focus on improving the treatment of AF and preventing its complications, such as stroke and heart failure. Thromboembolism, caused by irregular myocardial cell contraction, is frequently observed in patients with AF without the use of anticoagulants. It has been reported that patients with AF have a mortality risk from a stroke that is two times that of patients without AF (2). For this reason, patients with non-valvular atrial fibrillation (NVAF) are prescribed anticoagulation therapy based on their CHA2DS2-VASc score, which is essential for their treatment (3, 4).

Direct oral anticoagulants (DOACs) include factor Xa inhibitors and direct thrombin inhibitors (DTI). The former inhibits both the direct and indirect coagulation pathways by occupying the active site of the factor Xa molecule. DTI, as its name implies, acts directly on the prothrombin transformation process to prevent fibrin formation. Vitamin K antagonists (VKA) exert their pharmacological function by inhibiting vitamin K epoxide reductase (VKOR). This enzyme catalyzes the conversion of vitamin K to dihydroquinone, which is required for glutamic acid carboxylation (5, 6). Although anticoagulants reduce the incidence of thrombus formation in patients, the oral administration of warfarin to patients with AF may result in renal injuries and accelerated progression of chronic kidney disease (CKD), known as “warfarin-related nephropathy” (WRN) (7, 8). Some studies reported that DOAC users are less likely to experience unfavorable renal outcomes than warfarin users (9). In this systemic review, we aimed to compare the risk of AKI in patients with NVAF caused by agents in DOACs and VKA.

Previous meta-analyses have revealed that, compared with warfarin, using DOACs is linked to a lower risk of developing AKI (10). However, due to differences in the action mechanisms of these drugs, it might be inappropriate to evaluate their renal outcomes together. The drugs in the DOACs group were analyzed separately in this systemic review to provide clinicians with more accurate guidance when deciding which oral anticoagulants should be given to patients with NVAF who require anticoagulation therapy.

## Methods

**Figure 1:**
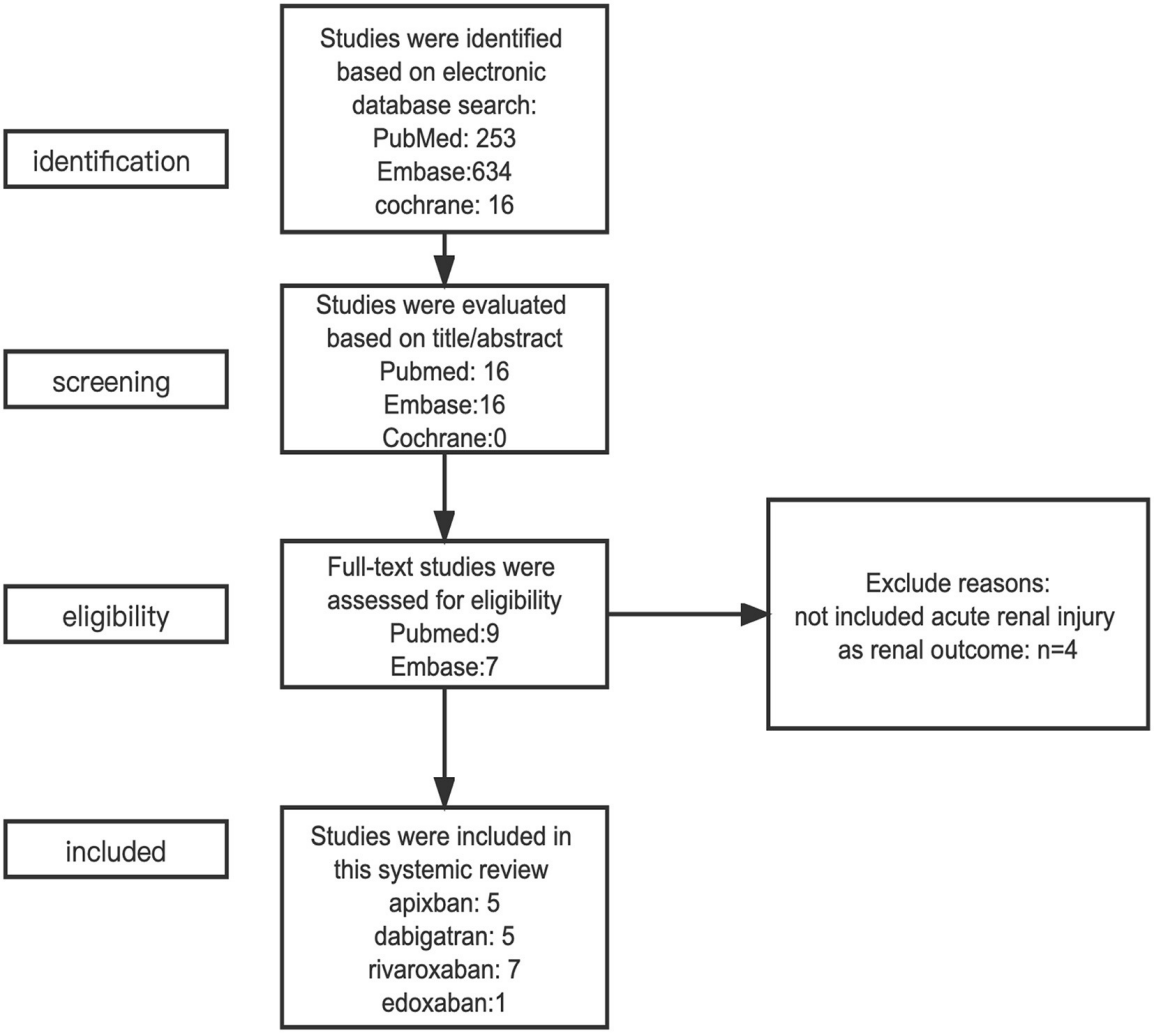
The process of selecting eligible studies.

We conducted an electronic search in PubMed, Embase, and the Cochrane Library. The restricted date range was from 1 January 2000 to 20 November 2022. We searched the database using keywords and free-text words on atrial fibrillation, acute kidney injury and oral anticoagulants. Following the search, strategies were applied: (acute kidney injury OR acute renal injury OR acute renal failure OR acute kidney failure OR warfarin related nephropathy OR AKI OR ARF OR WRN) AND (atrial fibrillation OR auricular atrial fibrillation OR non-valvular atrial fibrillation OR NVAF) AND (oral anticoagulants OR OAC OR warfarin OR vitamin K antagonist OR VKA OR non-vitamin K antagonist oral anticoagulant OR NOAC OR direct oral anticoagulant OR DOACs OR novel oral anticoagulant OR dabigatran OR apixaban OR rivaroxaban OR edoxaban). Then, the studies were assessed based on their title and abstract. Afterward, we performed a selection process based on the full texts of the literature. All the processes were conducted independently by two investigators. The process of selecting the studies is displayed in Figure 1.

## Eligibility criteria

Eligible studies must include patients with NVAF receiving anticoagulation therapy. The anticoagulants used by those patients are either DOACs or VKA. Studies focusing on comparing the difference in the risk of AKI induced by DOACs and warfarin were included. The included studies must be case-control studies, cohort studies, or randomized clinical trials (RCT). For all the included studies, the results need to be reported in the form of an odds ratio (OR), relative risk (RR), or hazard ratio (HR). Moreover, a clear definition of AKI needs to be given in the studies. All the studies included were written in English.

## Study selection

The eligibility of studies was determined through a review of their titles, abstracts, and full texts. Once selected, baseline information was extracted from these studies, including the author, year of publication, study design, characteristics of the study population, types of oral anticoagulants used, and results. This information was used to assess the suitability of the studies for inclusion in the analysis.

## Study quality assessment

To evaluate the overall quality of the research, the Newcastle–Ottawa tool was used to evaluate the quality of the included observational studies (11). Selection of the population, comparability of the included subjects, and study outcomes were considered when evaluating the included literature. Literature with seven or more stars was considered superior. For the randomized clinical trials, an assessment based on the Cochrane risk-of-bias tool was performed. The results are shown in Supplementary Tables 1, 2. The publication bias of all included studies was assessed by the Egger test (*P* = 0.091).

## Results

### Apixaban and acute kidney injury

Four observational studies and one randomized clinical trial comparing the effects of apixaban and warfarin on AKI risk in patients with NVAF were included in this review (12–16). The eligible studies included populations from different regions, such as Asia, North America, and Europe. All five studies assessed the risk of AKI as a renal outcome, while efficacy outcomes were only discussed in the RCT, which suggests that apixaban-treated patients have a significantly lower risk of stroke compared to the warfarin-treated population. The results of the included studies suggest that apixaban is associated with a reduced risk of AKI (Table 1). Subgroup analysis was performed in three studies based on renal function, and one study divided patients into two groups based on the presence of chronic kidney disease (CKD). Divergence from the general conclusion could be observed in patients with severe renal impairment. Jung-Im Shin et al. found that patients treated with apixaban were more likely to develop AKI than patients treated with warfarin (13). However, another study conducted by Ziv Harel et al. indicates they have a significantly lower risk of AKI compared to another group (15). For this reason, it is currently unclear whether apixaban is superior to warfarin in terms of AKI for those patients with severe renal dysfunction. On the basis of current studies, patients taking apixaban tend to have a lower risk of developing AKI among most strata of renal function. However, for those with severe kidney dysfunction, further studies are required.

**Table 1:** Summary of studies comparing the risk of AKI induced by apixaban and warfarin.

**Author**	**Medication**	**Study design**	**Baseline characteristics of investigated population**	**Definition of acute kidney injury**	**Renal outcome**	**Efficacy** **outcome**
Xiaoxi et al. (12)	Apixaban (*n =* 1883) Warfarin (*n =* 4185)	Retrospective cohort study	Patients with non-valvular atrial fibrillation in the United States Apixaban vs. Warfarin: age(yrs.): 72.9 vs. 73.2 eGFR (ml/min per 1.73 m^2^): 67.6 vs. 66.9 Women (%): 47.8 vs. 44.8 CHA_2_DS_2_-VASc: 4.0 vs. 4.2	Hospitalization or emergency department with a diagnosis code of AKI at the primary or secondary position	Acute kidney injury: Apixaban vs. Warfarin: HR = 0.84 95 %CI (0.66,1.07) *P =* 0.16 eGFR ≥60ml/min per 1.73 m^2^ Sample size: NR HR = 0.75 95% CI (0.49, 1.14) eGFR < 60 ml/min per 1.73 m^2^ Sample size: NR HR = 0.94 95% CI (0.71, 1.25)	NR
Shin et al. (13)	Apixaban (*n =* 1029) Warfarin (*n =* 1029)	Retrospective cohort study	Patients with non-valvular atrial fibrillation in the United States Age (mean, yrs.): 72 years eGFR (ml/min per 1.73 m^2^): 69 Women (%): 47	Hospitalization or emergency department with a diagnosis code of AKI (ICD-9 clinical modification code 584. x.) at the primary or secondary position.	Acute kidney injury: Apixaban vs. Warfarin: Overall: HR = 0.86, 95% CI (0.68, 1.10) *P =* 0.233 eGFR≥60 ml/min per 1.73 m^2^: Sample size: NR HR = 0.67, 95% CI (0.45, 1.00) *P =* 0.052 eGFR 30-59 ml/min per 1.73m^2^: Sample size: NR HR = 1.01, 95% CI (0.72,1.41) *P =* 0.956 eGFR < 30 ml/min per 1.73m^2^: Sample size: NR HR = 1.23, 95% CI (0.58,2.61) *P =* 0.593	NR
Chan et al. (14)	Apixaban (*n =* 5875) (std. dose 5mg b.i.d : 39% Low dose 2.5mg b.i.d : 61%) Warfarin (*n =* 21135)	Retrospective cohort study	Patients with non-valvular atrial fibrillation in Taiwan CKD-free cohort (Dabigatran vs. Warfarin) Age(mean): 71 vs. 70 Women (%): 43 vs. 43 CHA_2_DS_2_-VASc: 3.05 vs. 2.94 CKD cohort (Dabigatran vs. warfarin) Age(mean): 77 vs. 76 Women (%): 39 vs. 38 CHA_2_DS_2_-VASc: 3.99 vs. 3.99	The outcomes of AKI were defined as those who received a diagnosis coded as ICD-9- CM 580.X, 584.X, or 586 (until Jan 1, 2016) and ICD-10-CM N17.x (from Jan 1, 2016, to Dec 31, 2016) during hospitalization or an outpatient visit at least once.	Acute kidney injury: Apixaban vs. Warfarin: CKD-free cohort: Sample size: 4368 vs. 16,908 HR = 0.65, 95% CI (0.60,0.72) CKD cohort: Sample size: 1507 vs. 4227 HR = 0.50, 95% CI (0.45,0.56)	NR
Harel et al. (15)	Apixaban (*n =* 8217) Warfarin (*n =* 8383)	Retrospective cohort study	Canadian patients diagnosed with atrial fibrillation. apixaban vs. warfarin: (mean) age(yrs.): 80 vs. 80 eGFR (ml/min per 1.73 m^2^): 65 vs. 65 Female (%): 54.85 vs. 53.64 CHA_2_DS_2_-VASc: 4.0 vs. 4.0	Hospitalization or emergency department presentation with AKI defined as a ≥50% increase in serum creatinine concentration from baseline, an absolute increase of ≥0.3 mg/dl, or the receipt of acute dialysis	Acute kidney injury: Apixaban vs. Warfarin: Overall: HR = 0.79, 95% CI (0.66, 0.94) eGFR≥60 ml/min per 1.73 m^2^: sample size: 5007 vs.5128 HR = 0.97, 95% CI (0.73,1.12) eGFR 30-59 ml/min per 1.73m^2^: sample size: 3,025 vs. 3065 HR = 0.76, 95% CI (0.64,0.91) eGFR < 30 ml/min per 1.73m^2^: sample size: 158 vs. 189 HR = 0.54, 95% CI (0.35,0.83)	NR
Granger et al. (16)	Apixaban (*n =* 9,088, 5 mg b.i.d: 95.3% 2.5 mg b.i.d: 4.7%) Warfarin (*n =* 9,052 5 mg b.i.d: 95.6% 2.5 mg b.i.d: 4.4%)	Randomized clinical trials	Patients from North America, Latin America, Europe, and Asia Pacific diagnosed with atrial fibrillation. Apixaban vs. warfarin Age(yrs): 70 vs. 70 CHADS_2_ score: 2.1 vs. 2.1 The proportion of patients with moderate to severe renal impairment: 16.5% vs. 16.7%	NR	Acute kidney injury: RR:0.615 95% CI (0.403, 0.938)	Stoke: HR = 0.79 95% CI (0.65,0.95)

NR, not reported in the included studies; AKI, acute kidney injury; yrs, years; ICD-9, international classification of disease ninth revision; ICD-10, international classification of disease tenth revision; HR, hazard ratio; 95% CI, 95% confidence interval; std dose: standard dose; b.i.d: bis in die, twice daily; RR: risk ratio; eGFR, estimated glomerular filtration rate.

### Dabigatran and acute kidney injury

In this systemic review, five studies comparing the AKI risk induced by dabigatran and warfarin were included (Table 2) (12–15, 17). The efficacy outcomes were discussed in the study conducted by Connonlly et al., which revealed that high-dose dabigatran has a significantly lower risk of stroke, whereas such an advantage cannot be detected in low-dose therapy. Based on the overall results of the included studies, patients with NVAF have a lower risk of AKI when treated with dabigatran. All included studies suggest that dabigatran is associated with a reduced risk of AKI compared to warfarin, except for the one conducted by Connonlly et al. (17), which indicates that dabigatran shows no superiority over warfarin in terms of a decreased AKI risk, with RRs of 1.203 and 1.075 for the low dose and high dose groups, respectively. Based on the overall results of the included studies, patients with NVAF tend to have a lower risk of AKI when treated with dabigatran. Three of the eligible studies performed subgroup analyses of the AKI risk induced by dabigatran and warfarin in patients with different renal functions. One study (13) suggests that, for patients with severe renal dysfunction, dabigatran has an increased risk of AKI relative to warfarin, while another study (15) did not report the result due to the small sample size. According to the results of the present studies, it is unclear whether dabigatran-treated patients with severe renal deficiency could have a lower risk of AKI compared to the warfarin-treated population.

**Table 2:** Summary of studies comparing the risk of AKI induced by warfarin and dabigatran.

**Author**	**Medication**	**Study design**	**Baseline characteristics of the investigated population**	**Definition of acute kidney injury**	**Renal outcome**	**Efficacy outcome**
Xiaoxi et al. (12)	Dabigatran (*n =* 1216) Warfarin (*n* =4185)	Retrospective cohort study	Patients with non-valvular atrial fibrillation in the United States Dabigatran vs. warfarin: age(yrs.): 72.1 vs. 73.2 eGFR (ml/min per 1.73 m^2^): 67.8 vs. 66.9 Women (%): 43.0 vs. 44.8 CHA_2_DS_2_-VASc: 3.9 vs. 4.2	Hospitalization or emergency department with a diagnosis code of AKI at the primary or secondary position	Acute kidney injury: Apixaban vs. Warfarin: HR = 0.55 95 %CI (0.40,0.77) *p < * 0.001 eGFR ≥60ml/min per 1.73 m^2^ Sample size: NR HR = 0.63 95% CI (0.39, 1.03) eGFR < 60 ml/min per 1.73 m^2^ Sample size: NR HR = 0.54 95% CI (0.34, 0.84)	NR
Shin et al. (13)	Dabigatran (*n =* 852) Warfarin (*n =* 852)	Cohort study	Patients with non-valvular atrial fibrillation in the United States Age (mean): 72 years eGFR (ml/min per 1.73 m^2^): 69 Women (%): 47	Hospitalization or emergency department with a diagnosis code of AKI (ICD-9 clinical modification code 584. x.) at the primary or secondary position.	Acute kidney injury: Dabigatran vs. Warfarin: Overall: HR = 0.70 95% CI (0.52, 0.96) *P =* 0.025 eGFR≥60 ml/min per 1.73 m^2^: HR = 0.66 95% CI (0.43, 1.02) *P =* 0.063 eGFR 30–59 ml/min per 1.73m^2^: HR = 0.60, 95% CI (0.37,0.98) *P =* 0.040 eGFR < 30 ml/min per 1.73m^2^: HR = 1.52, 95% CI (0.53,4.38) *P =* 0.440	NR
Chan et al. (14)	Dabigatran, (*n =* 20145) (std. dose 150 mg b.i.d: 11% low dose: 110 mg b.i.d 89%) warfarin (*n =* 21135)	Retrospective cohort study	Patients with non-valvular atrial fibrillation in Taiwan CKD-free cohort (dabigatran vs. warfarin) Sample size: 16,945 vs. 16,908 Age(mean): 71 vs. 70 Women (%): 43 vs. 43 CHA_2_DS_2_-VASc: 3.05 vs. 2.94 CKD cohort (dabigatran vs. warfarin) Sample size: 3200 vs. 4227 Age(mean): 77 vs. 76 Women (%): 39 vs. 38 CHA_2_DS_2_-VASc: 3.99 vs. 3.99	The outcomes of AKI were defined as those who received a diagnosis coded as ICD-9- CM 580.X, 584.X, or 586 (until Jan 1, 2016) and ICD-10-CM N17.x (from Jan 1, 2016, to Dec 31, 2016) during hospitalization or an outpatient visit at least once.	Acute kidney injury: Dabigatran vs. Warfarin: CKD-free cohort: HR = 0.68, 95% CI (0.64,0.74) CKD cohort: HR = 0.54, 95% CI (0.49,0.59)	NR
Harel et al. (15)	Dabigatran, (*n =* 2,277) Warfarin (*n =* 2,269)	Retrospective cohort study	Canadian patients with non-valvular atrial fibrillation Dabigatran vs. warfarin: age(yrs.): 78 vs. 78 eGFR (ml/min per 1.73 m^2^): 69 vs. 69 Women (%): 44.56 vs. 44.05 CHA_2_DS_2_-VASc: 4 vs. 4	Hospitalization or emergency department presentation with AKI defined as a ≥50% increase in serum creatinine concentration from baseline, an absolute increase of ≥0.3 mg/dl, or the receipt of acute dialysis	Acute kidney injury: Dabigatran vs. Warfarin: Overall: HR = 0.54, 95% CI (0.43,0.69) eGFR≥60 ml/min per 1.73 m^2^: sample size: 1,535 vs. 1,543 HR = 0.69, 95% CI (0.52, 0.90) eGFR 30-59 ml/min per 1.73m^2^: sample size: 727 vs. 712 HR = 0.60, 95% CI (0.44,0.82) eGFR ≤ 30 ml/min per 1.73m^2^: NR	NR
Connonlly et al. (17)	Dabigatran (*n =* 12042. 110mg b.i.d : 5,983 150mg b.i.d: 6,059) Warfarin (*n =* 5998)	Randomized clinical trial	Patients from North America, Latin America, Europe, and Asia Pacific diagnosed with atrial fibrillation. Dabigatran (110mg b.i.d) vs. Dabigatran (150 mg b.i.d) vs. Warfarin: Age(yrs): 71.4 vs. 71.5 vs. 71.6 Women (%): 35.7 vs. 36.8 vs. 36.7 CHADS_2_ score: 2.1 vs. 2.2 vs. 2.1	NR	Acute kidney injury: Low-dose (110mg b.i.d) group: Dabigatran vs. warfarin RR = 1.203 95% CI (0.769, 1.881) High-dose (150 mg b.i.d) group: RR=1.075 95% CI (0.680, 1.699)	Stroke Low-dose (110mg b.i.d) group: Dabigatran vs. warfarin HR = 0.91 95% CI (0.74, 1.11) High dose (150 mg b.i.d) group: HR = 0.66 95% CI (0.53,0.82)

NR, not reported in the included studies; AKI, acute kidney injury; yrs, years; ICD-9, international classification of disease ninth revision; ICD-10, international classification of disease tenth revision; HR, hazard ratio; RR, risk ratio; 95% CI, 95% confidence interval; eGFR, estimated glomerular filtration rate; std dose: standard dose; b.i.d: bis in die, twice a day; CKD, chronic kidney disease.

### Rivaroxaban and acute kidney injury

This review included seven studies conducted in various regions, including Asia, North America, and Europe (12–15, 18–20). Only one study evaluated an efficacy outcome, which showed that the risk of stroke was similar between the low-dose rivaroxaban group and the warfarin group, while high-dose rivaroxaban therapy was associated with a significantly lower risk of stroke compared to warfarin (20). In comparison to warfarin, the overall results revealed that using rivaroxaban is associated with a lower risk of developing AKI (Table 3). Five studies included a subgroup analysis; four were based on the stratification of renal function, and one divided the cohorts into CKD and CKD-free groups. Yao et al. (12) suggested that, for patients with deficient renal function (eGFR < 60 ml/min per 1.73 m^2^), the AKI risk induced by rivaroxaban does not differ significantly from that of warfarin. Divergence from the general conclusion could be observed in the population with severe renal dysfunction; the study conducted by Jung-Im Shin et al. (13) indicated that rivaroxaban is related to a higher risk of AKI, while the results of the other two studies suggest that the risk of AKI associated with rivaroxaban is not significantly different from that of warfarin (15, 19). According to current studies, rivaroxaban may not be superior to warfarin in reducing AKI risk for NVAF patients with severely impaired renal function.

**Table 3:** Summary of studies comparing the risk of AKI induced by rivaroxaban and warfarin.

**Author**	**Medication**	**Study design**	**Baseline characteristics of the investigated population**	**Definition of acute kidney injury**	**Renal outcome**	**Efficacy outcome**
Xiaoxi et al. (12)	Rivaroxaban (*n =* 2,485) Warfarin (*n =* 4,185)	Retrospective cohort study	Patients with non-valvular atrial fibrillation in the United States Rivaroxaban vs. warfarin: age(yrs.): 72.3 vs. 73.2 eGFR (ml/min per 1.73 m^2^): 69.0 vs. 66.9 Women (%): 43.8 vs. 44.8 CHA_2_DS_2_-VASc: 3.9 vs. 4.2	Hospitalization or emergency department with a diagnosis code of AKI (ICD-10M) at the primary or secondary position.	Acute kidney injury: Rivaroxaban vs. Warfarin Overall: Sample size: 2,485 vs. 4185 HR = 0.69 95 %CI (0.57,0.84) *p < * 0.001 eGFR ≥60ml/min per 1.73 m^2^ Sample size: HR = 0.62 95% CI (0.44, 0.87) eGFR < 60 ml/min per 1.73 m^2^ Sample size: NR HR = 0.81 95% CI (0.63, 1.03)	NR
Shin et al. (13)	Rivaroxaban, (*n =* 1,325) Warfarin (*n =* 1,325)	Retrospective cohort study	Patients with non-valvular atrial fibrillation in the United States Age (mean): 72 years eGFR (ml/min per 1.73 m^2^): 69 Women (%): 47	Hospitalization or emergency department with a diagnosis code of AKI (ICD-9 clinical modification code 584. x.) at the primary or secondary position.	Acute kidney injury: Rivaroxaban vs. Warfarin: Overall: HR = 0.83 95% CI (0.66, 1.05) *P =* 0.114 eGFR≥60 ml/min per 1.73 m^2^: HR = 0.79 95% CI (0.50, 0.98) *P =* 0.037 eGFR 30-59 ml/min per 1.73m^2^: HR = 0.95 95% CI (0.68,1.33) *P =* 0.764 eGFR < 30 ml/min per 1.73m^2^: HR = 1.48 95% CI (0.63,3.49) *P =* 0.36	NR
Chan et al. (14)	Rivaroxaban, (*n =* 28,066) (std. dose: 93% Low dose: 7%) Warfarin (*n =* 21,135)	Retrospective cohort study	Patients with non-valvular atrial fibrillation in Taiwan CKD-free cohort (rivaroxaban vs. warfarin) Age(mean): 71 vs. 70 Women (%): 43 vs. 43 CHA_2_DS_2_-VASc: 3.04 vs. 2.94 CKD cohort (rivaroxaban vs. warfarin) Age(mean): 76 vs. 76 Women (%): 38 vs. 38 CHA_2_DS_2_-VASc: 3.99 vs. 3.99	The outcomes of AKI were defined as those who received a diagnosis coded as ICD-9- CM 580.X, 584.X, or 586 (until Jan 1, 2016) and ICD-10-CM N17.x (from Jan 1, 2016, to Dec 31, 2016) during hospitalization or an outpatient visit at least once.	Acute kidney injury: Rivaroxaban vs. Warfarin: CKD-free cohort: Sample size: 22,301 vs. 16,908 HR = 0.73 95% CI (0.68,0.79) CKD cohort: Sample size: 5765 vs. 4227 HR = 0.53 95% CI (0.49,0.58)	^NR^
Hernandez et al. (18)	Rivaroxaban (*n =* 10,017) (std dose 20mg o.d ,: 77.4% Low dose 15mg o.d: 22.6%). Warfarin (*n =* 11665)	Retrospective cohort study	Patients with non-valvular atrial fibrillation and a baseline history of type 1 or type 2 diabetes in the United States Age(median): 70 years CHA_2_DS_2_-VASc (median): 3	Based on the definition of acute kidney injury in ICD-10 code.	Acute kidney injury: Rivaroxaban vs. warfarin HR = 0.83 95% CI (0.74-0.92) Stage 3-4 CKD cohort: HR = 0.63 95% CI (0.49-0.79) No Stage 3-4 CKD: HR = 0.89 95% CI (0.78-1.00)	NR
Harel et al. (15)	Rivaroxaban (*n =* 5,263) Warfarin (*n =* 5,363)	Retrospective cohort study	Canadian patients with atrial fibrillation Rivaroxaban vs. warfarin: age(yrs.): 78 vs. 78 eGFR (ml/min per 1.73 m^2^): 70 vs. 70 Women (%): 51.97 vs. 50.39 CHA_2_DS_2_-VASc: 4 vs. 4	Hospitalization or emergency department presentation with AKI defined as a ≥50% increase in serum creatinine concentration from baseline, an absolute increase of ≥0.3 mg/dl, or the receipt of acute dialysis	Acute kidney injury: Rivaroxaban vs. Warfarin: Overall: HR = 0.77 95% CI (0.63, 0.93) eGFR≥60 ml/min per 1.73 m^2^: sample size: 3,777 vs. 3,898 HR = 0.94 95% CI (0.76, 1.14) eGFR 30–59 ml/min per 1.73 m^2^: sample size: 1,439 vs. 14,118 HR = 0.70 95% CI (0.57, 0.86) eGFR < 30 ml/min per 1.73m^2^: sample size: 47 vs. 47 HR = 0.83 95% CI (0.35,1.95)	NR
González-Pérez et al. (19)	Rivaroxaban (*n =* 6,436 Dose: 15/20 mg/day) Warfarin (*n =* 7,129)	Retrospective cohort study	Patients with non-valvular atrial fibrillation registered in IMRD-UK Rivaroxaban vs. warfarin: age(yrs.): 74.4 vs. 74.2 eGFR (ml/min per 1.73 m^2^): 70.5 vs. 70.4 Female (%): 42.7 vs. 43.7 CHA_2_DS_2_-VASc: 3.2 vs. 3.3	Based on recorded SCr values using validated Aberdeen AKI phenotyping. AKI is determined if any of following 3 criteria were met: 1) Serum creatinine ≥1.5 times higher than the median of all creatinine values 8-365 days ago. 2) Serum creatinine ≥1.5 times higher than the lowest creatinine within 7 days. 3) serum creatinine >26 μmol/L higher than the lowest creatine within 48 hours.	Acute kidney injury Rivaroxaban vs. warfarin Overall: HR = 0.80, 95%CI (0.68,0.93) eGFR>50 ml/min/1.73m^2^: Sample size: 5547 vs. 6043 HR = 0.79 95% CI (0.66,0.94) eGFR ≤ 50 ml/min/1.73m^2^: Sample size: 889 vs. 1,086 HR = 0.82 95% CI (0.59,1.14)	NR
Manesh R Patel et al. (20)	Rivaroxaban (*n =* 7,131, 20 mg o.d, 15 mg o.d. for patients with CrCL 30–49 ml /minute) Warfarin (*n =* 7,133, adjusted dose to target INR, 2.0 to 3.0)	Randomized clinical trials	Patients with non-valvular atrial fibrillation from 45 countries. Rivaroxaban vs. warfarin: 71.2 vs. 71.2 Female (%): 39.6 vs. 39.7	NR	Acute kidney injury: Rivaroxaban vs. warfarin: HR = 0.806 95% CI (0.523, 1.241)	Stroke Low-dose (110mg b.i.d) group: Dabigatran vs. warfarin HR = 0.91 95% CI (0.74, 1.11) High dose (150 mg b.i.d) group: HR = 0.66 95% CI (0.53,0.82)

NR, not reported in the included studies; AKI, acute kidney injury; yrs, years; ICD-9, international classification of disease ninth revision; ICD-10, international classification of disease tenth revision; HR, hazard ratio; RR, risk ratio; 95% CI, 95% confidence interval; eGFR, estimated glomerular filtration rate; SCr, serum creatinine; std dose: standard dose; o.d.:omni die, once daily; IMRD-UK: the IQVIA Medical Research Data UK, a database representative of UK general population.

### Edoxaban and acute kidney injury

A single randomized clinical trial (21) was conducted to analyze the effect of edoxaban on NVAF patients (Table 4). The study participants were divided into three groups based on the dosage and type of treatment they received. The results indicated that patients in both the low-dose edoxaban group and the high-dose edoxaban group had a slightly lower risk of AKI compared to the warfarin group. In terms of safety outcomes, the low-dose edoxaban group had a higher risk of stroke, while the high-dose group had a slightly lower, but not statistically significant, risk of stroke.

**Table 4:** Summary of studies comparing the risk of AKI induced by edoxaban and warfarin.

**Author**	**Medication**	**Study design**	**Baseline characteristics of investigated population**	**Definition of acute kidney injury**	**Renal outcome**	**Efficacy outcome**
Giugliano et al. (21)	Edoxaban, High dose (60mg o.d.): 7,012 Low dose (30 mg o.d.): 7,012 Warfarin: (Dose adjusted to achieve INR, 2.0 to 2.0)	Randomized clinical trial	Patients with non-valvular atrial fibrillation from Asia (13.7%), America (82.3%) Age (yrs): High-dose edoxaban vs. low-dose edoxaban vs. warfarin (median) 72 vs. 72 vs.72 Women (%): 37.9 vs. 38.8 vs. 37.5 CHADS_2_ score: 2.8 vs. 2.8 vs. 2.8	NR	Acute kidney injury: Low dose edoxaban vs. warfarin HR = 0.984 95% CI (0.694, 1.395) High dose edoxaban vs. warfarin HR = 0.841 95% CI (0.585, 1.210)	Stroke Low dose (30mg o.d) HR = 1.13 95% CI (0.97,1.31) High dose (60mg o.d.) HR = 0.88 95% CI (0.75,1.03)

NR, not reported in the included studies; HR, hazard ratio; RR: risk ratio; 95% CI, 95% confidence interval; o.d.: omni die, once daily; yrs, years.

The overall results suggest that DOACs, except for edoxaban, are associated with a lower risk of AKI than warfarin in patients with NVAF from various regions. Subgroup analysis based on eGFR stratification revealed that, for patients with severe renal deficiencies, DOACs may not provide a significantly lower risk of AKI compared to warfarin.

## Discussion

Six observational studies and four randomized clinical trials were included in our systemic review to compare the risk of AKI caused by warfarin and DOACs (rivaroxaban, dabigatran, apixaban, and edoxaban). Instead of being analyzed as a group, agents belonging to DOACs were analyzed individually. The results indicate that DOACs, except for edoxaban, are associated with a lower incidence of AKI compared to warfarin. The conclusion of this systemic review is consistent with that of prior analyses, which also focused on the renal outcomes of DOACs (10, 22, 23). The result of edoxaban might be caused by the lack of edoxaban-related studies. Subgroup analysis based on the stratification of renal function was also involved. The results demonstrate that DOACs may not be superior to warfarin in terms of AKI risks in patients with severe renal impairment.

Thrombin inhibition and vascular calcification caused by vitamin K deficiency may explain why populations treated with DOACs have a lower AKI incidence compared to warfarin-treated populations. Erythrocytes and the cast of red blood cells can be observed under a microscope in renal biopsies of patients with WRN, an injury that has long been demonstrated, indicating the presence of endothelial wall impairment (24, 25). The damage to endothelial wall integrity is assumed to be associated with thrombin since it participates in the maintenance of the endothelial barrier by activating protease-activated receptors (26–28). Moreover, another hypothesis suggests that vascular calcification induced by vitamin K deficiency is related to renal deterioration. Matrix G1α protein (MGP), a protein whose activation requires vitamin K-dependent carboxylase, can inhibit bone morphogenic protein-2 (BMP_2_), which can elevate the expression level of osteogenesis markers within a cell (29, 30). In the absence of sufficient vitamin K, the level of BMP_2_ rises, resulting in vascular calcification, which is believed to have a direct relationship with a decline in renal function (31).

The results also revealed that DOACs may not be superior to warfarin with respect to AKI risk for patients with severe renal deficiency. However, previous meta-analyses (32) revealed that patients treated with DOACs were less likely to experience renal deterioration than those treated with warfarin; this conclusion may not apply to those with severe renal dysfunction since patients with severe kidney dysfunction were excluded in the original studies (16, 17, 20, 21). The pharmacokinetics and pharmacodynamic properties of DOACs may account for the results in patients with severe renal dysfunction. Agents belonging to DOACs undergo renal elimination, with proportions being approximately 80%, 35%, 27%, and 50% for dabigatran, rivaroxaban, apixaban, and edoxaban, respectively. In contrast, warfarin molecules are not eliminated through the kidneys (33). In patients whose renal functions are severely compromised, using DOACs increases the burden on the kidney, resulting in acute deterioration of kidney function. Moreover, the improper usage of DOACs in clinical practice may also provide an explanation for the results. According to the guidelines, reduced doses of DOACs are recommended for patients with renal insufficiency (34). However, a survey conducted by Yao et al. (35) demonstrated that a large proportion of patients potentially overdosed, which contributes to undesired renal outcomes.

Among the observational studies included, only one study included the Asian population. Notably, compared to other populations, Asian populations are more likely to receive low-dose DOACs. This finding is consistent with a prior clinical trial that claimed that, for patients with NVAF in Asia, low-dose treatment is favored because of the relatively higher prevalence of CKD in the Asian population (36, 37). This can result in a lower risk of renal injury since the kidney injury induced by DOACs is dose-dependent (38). Moreover, Asian patients are reported to have poorer quality control, which can induce a higher risk of WRN compared to the non-Asian populations (36). Although the conclusion of the study conducted in the Asian population is consistent with that for other populations, further studies are required to analyze the risk of AKI among Asian NVAF populations taking a standard dose of DOACs.

Admittedly, there are some limitations to this review. First, the majority of the included studies are observational. In four of the included studies, instead of diagnosing AKI based on recorded data, the diagnoses of AKI were largely based on the physician's judgment in clinical practice since the codes that indicate AKI in the International Classification of Disease (ICD) are applied. In addition, we found in these studies that AKI can be caused by factors other than warfarin. Moreover, as WRN is dose-dependent, the daily dose of DOACs and time in the therapeutic range (TTR) of warfarin-treated patients, for instance, may influence the final results (38). The daily dose of the drugs and TTR were not reported in most of the eligible studies, which may introduce bias into the results. Second, efficacy outcomes were not evaluated in most of the included studies. Efficacy outcomes must be included to provide more detailed guidance so that physicians can make better decisions after weighing the pros and cons. In addition, most of the present studies are conducted in the non-Asian population. The only study conducted in an Asian population included a large proportion of patients taking low-dose DOACs. For this reason, whether the conclusion in this review can be applied to Asian populations taking a standard dose of DOACs remains unclear.

Despite these limitations, our review also has some advantages over the others, and it provides directions for future research and clinical practice. First, only one clinical trial focusing on edoxaban was included. With the increased application of edoxaban in clinical practice, more studies examining its efficacy and safety should be conducted. Second, our review suggests that more studies are required in patients with severe renal insufficiency. Given that AF and chronic kidney disease are two diseases with shared risk factors and have a bidirectional relationship with each other (39), clarifying the correlation between decreased kidney function and AKI risk induced by DOACs will assist doctors in making more accurate choices when selecting oral anticoagulants for their patients. Furthermore, a few of the current studies investigate the difference in AKI risk among DOACs. The agents in DOACs do not perform their roles with identical mechanisms. For example, dabigatran specifically acts on thrombin, while the other three act pharmacologically on coagulation factors. As discussed above, thrombin inhibition may also induce renal injury. Therefore, agents in DOACs may correlate to different AKI risks, and elucidating the variance among DOACs aids in more accurate usage of DOACs. In addition, the study conducted in Asian populations suggests that the usage of DOACs in Asians differs from that in other populations. More studies focusing on the efficacy and safety of DOACs should be conducted in this population. Indeed, low-dose DOACs provide a lower risk of AKI for Asian patients, but whether low-dose therapy has the same effect on reducing stroke risk remains unclear in the included study. Efficacy outcomes should also be included in future studies.

Except for providing potential directions for further study, our findings also provide directions for clinical practice. For patients with normal or relatively preserved renal function, DOACs are favored since they can provide a lower risk of AKI. However, for patients with severe renal insufficiency, the use of anticoagulants needs to be individualized, and comprehensive evaluations are required before deciding the agent and its dosage for the patient. The results also indicate that renal functions should be routinely monitored when anticoagulants are prescribed to patients. The dose of DOACs should be adjusted immediately after the detection of severe renal insufficiency since an overdose of DOACs induces a higher risk of renal impairment in those patients.

## Conclusion

Patients with NVAF who are treated with DOACs have a lower risk of developing AKI compared to those treated with warfarin. However, the effects of DOACs on patients with impaired renal functions remain unclear, and more studies are required to determine whether they are better options for those with severe renal dysfunction.

## Data availability statement

The original contributions presented in the study are included in the article/Supplementary material, further inquiries can be directed to the corresponding author.

## Author contributions

All authors listed have made a substantial, direct, and intellectual contribution to the work and approved it for publication.
