# Supplementary material for: Effects of direct oral anticoagulants vs. vitamin K antagonists on acute kidney injury in patients with atrial fibrillation: A systematic review
Source: Front Cardiovasc Med. 2023 Jan 26;10:1068269. doi: 10.3389/fcvm.2023.1068269 (PMC9909185; doi:10.3389/fcvm.2023.1068269)
Supplement: Supplementary file 1 [file Table_1.DOCX]

| **Cohort study** | **Selection** | | | **comparability** | | **Outcome** | | | **Quality score** |
| --- | --- | --- | --- | --- | --- | --- | --- | --- | --- |
|  | Representativeness of the exposed cohort | Selection of the non-exposed cohort | Ascertainment of exposure | Demonstration that outcome of interest was not present at start of study | Comparability of cohorts based on the design or analysis | Assessment of outcome | Was follow-up long enough for outcomes to occur | Adequacy of follow up of cohorts |  |
| Xiaoxi Y et al. (2017) | ★ | ★ | ★ | ☆ | ★★ | ★ | ★ | ★ | 8 |
| Jung-Im Shin et al. (2018) | ★ | ★ | ☆ | ☆ | ★★ | ★ | ★ | ★ | 7 |
| YI-Hsin Chan et al. (2018) | ★ | ★ | ★ | ☆ | ★★ | ★ | ★ | ★ | 8 |
| Ziv Harel et al. (2021) | ★ | ★ | ★ | ☆ | ★★ | ★ | ★ | ★ | 8 |
| Adrian V. Hernandez et al. (2020) | ★ | ★ | ★ | ☆ | ★★ | ★ | ★ | ★ | 8 |
| Antonio González-Pérez et al. (2022) | ★ | ★ | ★ | ★ | ★★ | ★ | ★ | ★ | 9 |

**Supplementary table 1**.Quality of cohort studies included in the review assessed by Newcastle-Ottawa quality assessment.


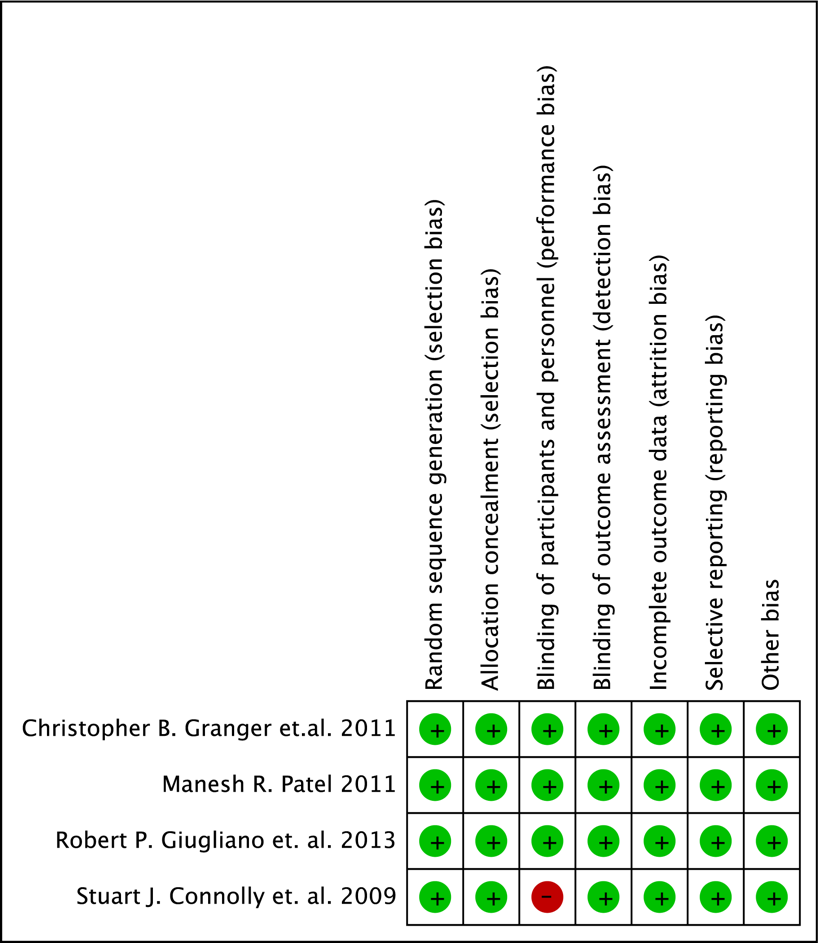


**Supplementary Table 2.** Quality of Randomized Clinical Trials assessed by using Cochrane risk-of-bias tool.
